# A data value metric for quantifying information content and utility

**DOI:** 10.1186/s40537-021-00446-6

**Published:** 2021-06-05

**Authors:** Morteza Noshad, Jerome Choi, Yuming Sun, Alfred Hero, Ivo D. Dinov

**Affiliations:** 1 Department of Electrical Engineering and Computer Science, University of Michigan, Ann Arbor, MI 48109 USA; 2 Statistics Online Computational Resource, University of Michigan, Ann Arbor, MI 48109 USA; 3 Department of Statistics, University of Michigan, Ann Arbor, MI 48109 USA; 4 Department of Biostatistics, University of Michigan, Ann Arbor, MI 48109 USA; 5 Department of Biomedical Engineering, University of Michigan, Ann Arbor, MI 48109 USA; 6 Michigan Institute for Data Science, University of Michigan, Ann Arbor, MI 48109 USA; 7 Stanford Center for Biomedical Informatics Research, Stanford University, Stanford, CA 94305 USA

**Keywords:** Data energy, Artificial intelligence, Machine learning, Data utility, Information content

## Abstract

Data-driven innovation is propelled by recent scientific advances, rapid technological progress, substantial reductions of manufacturing costs, and significant demands for effective decision support systems. This has led to efforts to collect massive amounts of heterogeneous and multisource data, however, not all data is of equal quality or equally informative. Previous methods to capture and quantify the utility of data include value of information (VoI), quality of information (QoI), and mutual information (MI). This manuscript introduces a new measure to quantify whether larger volumes of increasingly more complex data enhance, degrade, or alter their information content and utility with respect to specific tasks. We present a new information-theoretic measure, called Data Value Metric (DVM), that quantifies the useful information content (energy) of large and heterogeneous datasets. The DVM formulation is based on a regularized model balancing data analytical value (utility) and model complexity. DVM can be used to determine if appending, expanding, or augmenting a dataset may be beneficial in specific application domains. Subject to the choices of data analytic, inferential, or forecasting techniques employed to interrogate the data, DVM quantifies the information boost, or degradation, associated with increasing the data size or expanding the richness of its features. DVM is defined as a mixture of a fidelity and a regularization terms. The fidelity captures the usefulness of the sample data specifically in the context of the inferential task. The regularization term represents the computational complexity of the corresponding inferential method. Inspired by the concept of information bottleneck in deep learning, the fidelity term depends on the performance of the corresponding supervised or unsupervised model. We tested the DVM method for several alternative supervised and unsupervised regression, classification, clustering, and dimensionality reduction tasks. Both real and simulated datasets with weak and strong signal information are used in the experimental validation. Our findings suggest that DVM captures effectively the balance between analytical-value and algorithmic-complexity. Changes in the DVM expose the tradeoffs between algorithmic complexity and data analytical value in terms of the sample-size and the feature-richness of a dataset. DVM values may be used to determine the size and characteristics of the data to optimize the relative utility of various supervised or unsupervised algorithms.

## Introduction

### Background

Big data sets are becoming ubiquitous, emphasizing the importance of solving the challenge of balancing information utility, data value, resource costs, computational efficiency, and inferential reliability [1]. This manuscript tackles this problem by developing a new measure, called the Data Value Metric (DVM), that quantifies the energy, or information content, of large and complex datasets, which can be used as a yardstick to determine if appending, expanding, or otherwise augmenting the data size or complexity may be beneficial in specific application domains. In practice, DVM provides a mechanism to balance, or tradeoff, a pair of competing priorities (1) costs or tradeoffs associated with increasing or decreasing the size of heterogeneous datasets (sample size) and controlling the sampling error rate, and (2) expected gains (e.g., decision-making improvement) or losses (e.g., decrease of precision or variability increase) associated with the corresponding scientific inference. The computational complexity of the DVM method is directly proportional to that of calculating mutual information, which is linear in terms of the data size. Thus, the DVM complexity is determined directly by the inferential method or technique used to obtain the classification, regression, or clustering results, which may itself be non-linear. Hence, DVM calculations do not add significant overhead to the standard analytical protocol.

Although several performance measures exist for supervised and unsupervised inference tasks, it is difficult to use established methods to infer the sufficiency of the data for each specific inferential task. For example, one could use accuracy measures for a classification task. Assume that the accuracy of $70\%$ is achieved for a non-random, non-stationary, or non-homogeneous dataset. Then, the question is whether we can expect an increase of the accuracy by adding more samples or more features, or maybe use alternative models to increase the value of the resulting inference. In general, such questions are difficult to answer solely by considering a particular measure of performance on a given dataset. Several of the previous approaches measuring the quality of data are summarized below.

### Related work

Several previous studies have proposed metrics for assessing the information gain of a given dataset. For example, value of information (VoI) analysis, originally proposed in [2] with overviews in [3–5], is a decision-theoretic statistical framework representing the expected increased inference accuracy or reduction in loss based on additional prospective information [6]. The basic three types of VoI methods include (1) inferential and modeling cases for linear objective functions under simplified parameter distribution restrictions, which limits their broad practical applicability [3, 7]; (2) methods for estimating the *expected value of partial perfect information (EVPPI)* involving partitioning of the parameter space into smaller subsets and assuming constant and optimal inference over the local neighborhoods, within subsets [8, 9]; and (3) Gaussian process regression methods approximating the expected inference [10–12]. More specifically, for a particular parameter $\phi$, the EVPPI is the expected inferential gain, or reduction in loss, when $\phi$ is perfectly estimated. As the perfect $\phi$ is unknown in advance, this reduction of loss expectation is taken over the entire parameter space $\phi \in \Phi$:

$$\begin{aligned} EVPPI(\phi )= E_{\theta } (L(d^*,\theta )) - E_{\phi } ( E_{\theta | \phi } (L(d^*_{\phi }, \theta )) ), \end{aligned}$$

where *d* is decision, inference or action, $d^*_{\phi }$ is the optimal inference obtained when $\phi$ is known, $\theta$ is the the model parameter vector, *E* is the expectation, and $L(d,\theta )$ is the likelihood function [6]. Note that VoI techniques are mainly suitable for specific types of problems, such as evidence synthesis in the context of decision theory. Further, their computational complexity tends to be high and require nested Monte Carlo procedures.

Another relevant study [13] utilizes a unique decomposition of the differences (errors) between theoretical (population) parameters and their sample-driven estimates (statistics) into three independent components. If $\theta$ and ${\hat{\theta }}$ represent a theoretical characteristic of interest (e.g., population mean) and its sample-based parameter estimate (e.g., sample arithmetic average), respectively, then, the error can be canonically decomposed as:

$$\begin{aligned} \underbrace{\theta -{\hat{\theta }}}_{\text {error}} = \underbrace{A}_{\text {Data}\atopwithdelims ()\text {Quality}} + \underbrace{B}_{\text {Data}\atopwithdelims ()\text {Quantity}} + \underbrace{C}_{\text {Inference}\atopwithdelims ()\text {Problem Complexity}}. \end{aligned}$$

Suppose *J* is a (uniform) random subset indexing a sample from the entire (finite, *N*) population. For a sample $\{X_i: j\in I_n \}$, $R_j$ is a random-sample indicator function (with values 0 or 1) capturing whether $j\in I_n$. Of course, $\sum _{j=1}^N {R_j}=n$, *X* is a multidimensional design matrix capturing the attributes of the data (features), $g:X \longrightarrow \mathfrak {R}$ is a linking map that allows us to compute on samples (e.g., polynomial functions for moment calculations or indicator functions for distribution functions), $g_j=g(X_j)$ is a mapping of the *j*-th feature, $A=A(g,R)$ is a measure of association between $R_J$ and $G_J$, the sampling rate $f=E_J(R_J)=\frac{n}{N}$ (ratio of sample-to-population size), $B=\sqrt{\frac{1-f}{f}}$, and *C* is a measure encoding the difficulty of estimating the sample-based parameters (${\hat{\theta }}$).

Bayes error rate is another metric that quantifies the intrinsic classification limits. In classification problems, the Bayes error rate represents the minimal classification error achieved by any classifier [14, 15]. The Bayes error rate only depends on the distributions of the classes and characterizes the minimum achievable error of any classifier. Several previous studies proposed effective estimation methods for the Bayes error rate [14–17]. In particular, [18] obtains a rate-optimal non-parametric estimator of the Bayes error rate. The Bayes error rate may not be attainable with a practical classifier.

The proposed data value metric addresses the problem of measuring and tracking data information content relative to the intrinsic limits within the context of a specific analytical inferential model.

### Data value metric

For a given dataset, the information-theoretic definition of DVM employs mutual information (MI) [19, 20] to quantify the inferential gain corresponding to increasing the data size or the richness of its features. In general, mutual information evaluates the degree of relatedness between a pair of data sets. In particular, MI may be used to assess the information gain between an initial data set and its augmented counterpart representing an enhanced version of the former. When both random variables *X* and *Y* are either discrete or continuous, the mutual information can be defined by:

1 $$\begin{aligned} \begin{array} { c } { { \text {discrete} \atopwithdelims ()\text {distributions} }\ \ I ( X ; Y ) = \displaystyle \sum _ { \{ y \in Y , x \in X \} } p ( x , y ) \log \left( \frac{ p ( x , y ) }{ p ( x ) p ( y ) } \right) } \\ { { \text {continuous} \atopwithdelims ()\text {distributions} }\ \ I ( X ; Y ) = \displaystyle \int _{\{ y \in Y, x \in X \} } p(x, y ) \log \left( \frac{p (x, y)}{p(x) p(y) } \right) d x d y ,} \end{array} \end{aligned}$$

where *p*(*x*) and *p*(*y*) are the marginal probability distribution functions and *p*(*x*, *y*) is the joint probability function of *X* and *Y*. The non-negative and symmetric MI measure expresses the intrinsic dependence in the joint distribution of *X* and *Y*, relative to the assumption of *X* and *Y* independence. Thus, MI captures the *X* and *Y* dependence in the sense that $I(X; Y)=0$ if and only if *X* and *Y* are independent random variables, and for dependent *X* and *Y*, $I(X; Y)>0$. Further, the conditional mutual information is defined as follows:

2 $$\begin{aligned} I(X ; Y \mid Z)=I(X ; Y, Z)-I(X ; Z). \end{aligned}$$

DVM relies on a low-dimensional representation of the data and tracks the quality of the extracted features. Either the extracted features or the predicted values from a model can be used in a low-dimensional representation in the DVM formulation. For each dataset, the DVM quantifies the performance of a specified supervised or unsupervised inference method. The DVM formulation is inspired by the concept of information bottleneck in deep neural networks (DNNs) [21, 22]. Information bottleneck represents the trade-off between two mutual information measures: *I*(*X*; *T*) and *I*(*T*; *Y*), where *X* and *Y* are respectively the input and output of the deep learning model and *T* is an intermediate feature layer.

Instead of simply computing sample-driven parameter estimates, the DVM approach examines the information-theoretic properties of datasets relative to their sample-sizes, feature-richness, and the algorithmic complexity of the corresponding scientific inference. There are both similarities and differences between DMV and other VoI metrics. The main difference is that for model-based inference, some VoI metrics may have known, exact, or asymptotic expectations based on exact, or Markov chain Monte Carlo (MCMC), posterior estimates [23–25]. Whereas, under model-free inference, estimating the DVM theoretical or ergodic properties is difficult, in general. This challenge prevents the derivation of an exact linear decomposition of the error between population characteristics and their sample-driven counterparts.

This manuscript is organized as follows. In "Methods" section , we define the data value metric (DVM) as an information-theoretic function of the (training and testing) data and the specific inferential technique. This section also includes the computational details about an effective mutual information (MI) estimator and ensemble dependency graph estimator (EDGE) [22] as well as the implementation details of a DVM Python package we built, validated, and openly shared. A feature selection application of DVM is also discussed in this section. The estimation of the mutual information using the ensemble dependency graph estimator (EDGE) is discussed in "Mutual information estimation" section. "Results" section  includes experimental results illustrating the behavior of the proposed DVM metric on a wide range of real and simulated data, low- and high-energy signals, feature-poor and feature-rich datasets. "Conclusion and discussion" section summarizes the conclusions and provides a discussion about applications, possible improvements, limitations, and future work. In the Appendix, we provide DVM implementation details, source code references, additional results, and references to interactive 3D plots of DVM performance on real and simulated data.

## Methods

There are a wide range of artificial intelligence, machine learning, and statistical inference methods for classification, regression and clustering [1, 26–28]. The DVM metric is applicable to unsupervised and supervised, model-based and model-free approaches. We employed the following supervised classification methods to identify, predict, or label predefined classes, linear models [29, 30], random forest [31], adaptive [32] and gradient [33] boosting, and k-nearest neighbors [34]. In addition, we tested several unsupervised clustering approaches for categorizing and grouping objects into subsets without explicit a priori labels, K-means [35], Affinity Propagation [36], and Agglomerative clustering [37].

The data value metric (DVM) technique utilizes MI to quantify the energy of datasets relative to the corresponding inferential technique applied to interrogate the data. Our approach is based on transforming the triple (*T*, *S*, *g*), representing the training (model estimation) dataset, the testing (validation) dataset, and the specific inferential method, respectively, into random variables $X=g(X_T,X_S)$ and $Y=Y_S$ whose MI captures the data-method information content in the triple.

Depending upon the type of the intended inference on the data, we will define the DVM separately for *supervised* modeling and for *unsupervised* clustering. While the two definitions are congruent, this dichotomy is necessary to provide explicitly constructive definitions that can be used for a wide range of domain applications. Expanding the general regularization problem formulation, given a dataset, *D*, the DVM is defined as a mixture blending a fidelity term, *F*(*D*), and a regularization term, *R*(*D*):

3 $$\begin{aligned} DVM(D)=\underbrace{F(D)}_{\text {fidelity}} -\ \underbrace{\lambda }_{\text {penalty}} \underbrace{R(D)}_{\text {regularizer}}. \end{aligned}$$

The DVM fidelity term captures the usefulness of the sample data for the specified inferential task (supervised or unsupervised). The second, regularization, term penalizes the DVM based on the computational complexity of the corresponding inferential method. Thus, broadly speaking, the DVM depends on the data (including both training and testing sets) as well as the data-analytic technique used to obtain the desired inference.

Let’s first explain the rationale behind mixing fidelity and regularization in the DVM definition. Consider a case-study where a high-energy (low-noise) dataset provides sufficient information to derive either good prediction accuracy, for supervised modeling, or obtain stable clustering results, for unsupervised inference. Expanding heterogeneous data by either appending the number of samples or expanding the set of features may not always increase the DVM and may add substantial costs associated of collecting, managing, quality control, and processing the larger datasets. The penalty term in the DVM accounts for some of these potential detrimental effects due to inflating the data. The effect of the regularization term is mediated by the size of the penalty coefficient $\lambda$, which controls the DVM balance between quality of the inference and the algorithmic complexity. There are many possible alternative forms of the regularizer term, *R*(*D*), such as runtime, computational complexity, or computing costs. In our experiments, we use the Big-O computational complexity of training the predictor to quantify the regularization penalty term $R(D)= f(n)$. Table 1 shows the computational complexities of several commonly used classification (C) and regression (R) classifiers. The table uses the following notation: *n* represents the size of the training sample, *p* is the number of features, $k_{trees}$ is the number of trees (for tree-based classifiers), $m_{sv}$ is the number of support vectors (for SVM), and $o_{l_i}$ is the number of neurons at layer *i* in a deep neural network classifier.

**Table 1:** Computational complexity of several commonly used regression and classification techniques

Classifier	Type	Training	Prediction	
Linear Regression	*R*	${O(p^{2} n + p^{3} )}$	${O(p)}$	(4)
Decision Trees	C&R	${O(n^{2} p)}$	${O(p)}$	
Random Forest	C	${O(n^{2} pk_{{trees}} )}$	${O(pk_{{trees}} )}$	
Gradient Boosting	C&R	${O(npk_{{trees}} )}$	${O(pk_{{trees}} )}$	
SVM	C&R	${O(n^{2} p + n^{3} )}$	${O(m_{{sv}} p)}$	
k-Nearest Neighbors	C&R	*varies*	${O(np)}$	
Neural Networks	C&R	*varies*	${O(\sum _{i} o_{{l_{i} }} o_{{l_{{i + 1}} }} )}$	
Naive Bayes	C	${O(np)}$	${O(p)}$	

Next, we will focus solely on the more complex DVM fidelity term, which will be defined separately for the two alternative approaches-supervised prediction and unsupervised clustering.

### Representation of the fidelity term in low-dimensions

First we will define the DVM fidelity term based on low-dimensional representations of the data. The motivation behind this definition of the fidelity is driven by the neural networks (NNs) process of optimizing an objective function and identifying feature contributions. Let *X*, *T* and *Y* respectively denote the NN input layer, an intermediate feature layer, and the output layer.

In [21, 22], the mutual information measures *I*(*X*; *T*) and *I*(*T*; *Y*) are used to demonstrate the evolution of training in deep neural networks. *I*(*T*; *Y*) represents how the trained feature layer *T* is informative about the label. In the training process of a deep neurals network (DNN), *I*(*T*; *Y*) keeps increasing [21, 22]. On the other hand, *I*(*X*; *T*) shows the complexity of the representation *T*. In DNN, *I*(*X*; *T*) increases in the first training phase and it decreases in the compression phase [21, 22]. Thus, *T* is a good representation of *X* if its information about *Y* is maximized for a constrained complexity. This is equivalent to maximizing the following information bottleneck (*IB*) loss function [38]:

5 $$\begin{aligned} IB = I(T;Y) - \beta I(X;T), \end{aligned}$$

where $\beta$ is a Lagrange multiplier with the condition $\beta >0$.

The DVM formulation is inspired by the NN definition of information bottleneck loss function in equation (5). Intuitively, a feature vector *T* has high quality if it is informative about the label and its representation complexity is small. Thus, *IB* might be used as a measure of feature quality.

However, there are also problems with considering *IB* as a feature quality measure. First, in general, *IB* has no fixed range and it’s not a priori clear what values of *IB* represent high and low salient features. Second, the penalty term in the *IB* function, *I*(*X*; *T*), represents the information of the feature *T* about *X*, which captures both necessary and unnecessary information in order to predict *Y*. It may be better to only consider the information that is independent of *Y* as a penalty term. In terms of information theoretic measures, one could formulate this as conditional information *I*(*X*; *T*|*Y*). Note that this penalty term is minimized when the representation *T* yields the information of *Y* without extra information about *X*. An example of this case is when *Y* is an invertible function of *T*.

Thus, the proposed fidelity term for the *Data Value Metric (DVM)* is defined in terms of the mutual information and conditional mutual information measures introduced in (1) and (2) as follows:

6 $$\begin{aligned} \underbrace{F(T)}_{\text {DVM Fidelity}} = \frac{I(T;Y) - \beta I(X;T|Y)}{I(X;Y)}. \end{aligned}$$

The following remarks include some of the properties of the proposed fidelity measure.

#### Remark 1.a

The following inequality holds

7 $$\begin{aligned} I(T;Y) - \beta I(X;T|Y)\le I(X;Y), \end{aligned}$$

and the fidelity term of the DVM always has the following upper bound:

8 $$\begin{aligned} F(T)=\frac{I(T;Y) - \beta I(X;T|Y)}{I(X;Y)}\le 1. \end{aligned}$$

#### Remark 1.b

$F(T)=1$ if and only if the following equations are true:

9 $$\begin{aligned} I(X ; Y \mid T)=0, \end{aligned}$$

10 $$\begin{aligned} I(X ; T \mid Y)=0 \end{aligned}$$

The proof for the Remarks 1.a and 1.b is given in Appendix 1.

#### Remark 2

The fidelity term of the DVM can be simplified to the form of the standard information bottleneck [38]:

11 $$\begin{aligned} F(T)=\frac{I(Y;T) - \beta I(T;X|Y)}{I(X;Y)}&= \frac{I(Y;T) - \beta \left( I(T;X)-I(T;Y)\right) }{I(X;Y)}\nonumber \\&= \frac{(1+\beta ) I(Y;T) - \beta I(T;X)}{I(X;Y)} . \end{aligned}$$

**Fig. 1:**
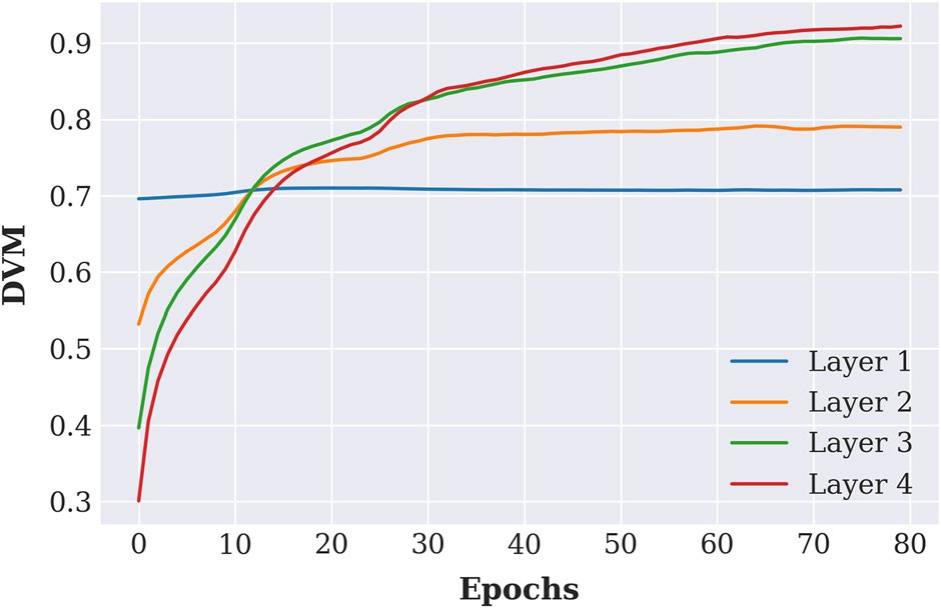
Training a neural network of size $784-200-100-60-30-10$ on the MNIST dataset with ReLU activation and using the DVM to track feature quality measures for different network layers. As shown, the deeper layers have higher DVM values, suggesting that they represent salient features for predicting the class labels

As a simple demonstration of the behavior of $DVM=F-\lambda R$, we fit a 5-layer DNN to predict the 10 class labels of the MNIST dataset [39], and used the DVM to track the feature quality across epochs and layers. The results of the DVM performance on the digit recognition is given Fig. 1. Since the network is trained as a whole with all layers, the regularizer term *R* is considered fixed for all layers. At a fixed training epoch, the DVM values in different network layers represent the trade-off between the information about the labels and the information about the input. These complementary information components are the first and second terms in the numerator of the DVM fidelity (11). During the iterative network training process, the information about the labels and the fidelity term increase, which suggests improvement of the quality of the feature layers.

### Supervised modeling

The DVM fidelity term definition, equation (6), relies on low-dimensional representations. Using the supervised-model predicted values, we can obtain low-dimensional representations that can be used to measure data quality in both supervised and unsupervised problems. Unsupervised inference problems will be considered later.

In supervised inference, we assume that we have a set of independent and identically distributed (i.i.d.) samples $X_i, 1\le i \le n$ with a joint distribution *f*(*x*) and associated known labels $Y_i$. We define encoder-decoder pair $({\mathcal {E}}, {\mathcal {D}})$, where ${\mathcal {E}}$ maps the high-dimensional input *X* into a lower dimensional representation *T*, and ${\mathcal {D}}$ maps the representation *T* to the predicted labels. In practice, we can think of ${\mathcal {E}}$ as a dimensionality-reduction method, or the intermediate representations of a deep neural network. In addition, ${\mathcal {D}}$ performs the classification task based on the lower dimensional representations. Note that if *T* is simply the predicted labels, the fidelity would depend on the specific classifier. However, if *T* is some low-dimensional representation of the data, such as extracted features or any intermediate layer of a deep neural network, the fidelity would be independent from the classifier and would only depend on the encoder (feature extraction) method.

The definition of the fidelity measure is based on a cross-validation type average of the definition (6) using the estimated mutual information measures. Given any random variables *X*, *Y* and *Z*, with corresponding sets of i.i.d. samples $\mathbf{X },\mathbf{Y }$ and $\mathbf{Z }$, $I(\mathbf{X };\mathbf{Y })$ denotes the estimated mutual information using the sample sets $\mathbf{X },\mathbf{Y }$.

We randomly split the feature set $\mathbf{X }$ into two subsets $(\mathbf{X }',\widetilde{\mathbf{X }})$. The first subset $(\mathbf{X }')$ is used for training, whereas the second one $(\widetilde{\mathbf{X }})$ is used for independent testing and *validation*. Also let $\widetilde{\mathbf{T }}$ denote the set of intermediate representation (or predicted labels), and $\widetilde{\mathbf{Y }}$ represent the true labels associated with the test dataset $\widetilde{\mathbf{X }}$. Then, we can define the DVM *fidelity* term by:

12 $$\begin{aligned} F:= \frac{1}{M}\displaystyle \sum _{i=1}^M {\frac{I(\widetilde{\mathbf{T }}_i;\widetilde{\mathbf{Y }}_i)- \beta I(\widetilde{\mathbf{X }}_i;\widetilde{\mathbf{T }}_i|\widetilde{\mathbf{Y }}_i)}{I(\mathbf{X };\mathbf{Y })}} . \end{aligned}$$

Using a weight coefficient, $\beta$, this fidelity term formulation, equation (12), mixes two components, $I(\widetilde{\mathbf{T }}_i;\widetilde{\mathbf{Y }}_i)$ and $I(\widetilde{\mathbf{X }}_i;\widetilde{\mathbf{T }}_i|\widetilde{\mathbf{Y }}_i)$, via normalization by $I(\mathbf{X };\mathbf{Y })$.

The first term, $I(\widetilde{\mathbf{T }}_i;\widetilde{\mathbf{Y }}_i)$, accounts for the fidelity of the low dimensional representation of the output labels, $\widetilde{\mathbf{Y }}_i$, whereas the second (penalty) term, $I(\widetilde{\mathbf{X }}_i;\widetilde{\mathbf{T }}_i|\widetilde{\mathbf{Y }}_i)$, accounts for the compression of the lower-dimensional representation.

The pseudo code below (Algorithm 1) outlines the computational implementation strategy we employ in the DVM package for evaluating the DVM. The metric captures the relative analytical value of the dataset relative to the computational complexity of the supervised prediction, classification, or regression problem. In practice, the regularization term, *R*(*g*), is estimated according to the known algorithmic complexity, see Table 1.

- **Input : ** Data sets $\mathbf{X }$, $\mathbf{Y }$, model *g*, parameters $\beta$, $\lambda$
- **for** *a random split* $(\mathbf{X }'_i,\widetilde{\mathbf{X }}_i)$ *of* $\mathbf{X }$ **do**
  - train *g* based on $(\mathbf{X }'_i,\mathbf{Y }'_i)$
  - $$\widetilde{\mathbf{T }}_i\leftarrow g(\widetilde{\mathbf{X }}_i)$$
  - $$F_i\leftarrow \frac{I(\widetilde{\mathbf{T }}_i;\widetilde{\mathbf{Y }}_i)- \beta I(\widetilde{\mathbf{X }}_i;\widetilde{\mathbf{T }}_i|\widetilde{\mathbf{Y }}_i)}{I(\mathbf{X };\mathbf{Y })} $$
- $$\widehat{D} \leftarrow \frac{1}{M} \sum _{i=1}^M F_i - \lambda R(g)$$
- **Output :** $$\widehat{D}$$

Algorithm 1: DVM calculation for supervised problems.

### Feature selection

Since DVM can be used to measure the quality of a feature set *T*, it can also serve as a feature selection method. In this section, we demonstrate a heuristic algorithm for sequential feature selection based on DVM values.

For a classification problem, the feature selection is defined as follows. Based on an initial feature set, choose a smaller set of features that yields a minimum prediction error. Let $X=\{X^1,...,X^d\}$ denote the *d* initial features. The objective is to select a smaller set of *r* features with maximum DVM score. One specific approach is based on a forward selection involving *r* iterative steps. At each step, we select a feature from the initial feature set, $\{X^1,...,X^d\}$, which increases DVM score the most. For a given (initial or intermediate) feature set ${{\mathcal {F}}}$, $DVM\{{{\mathcal {F}}}\}$ represents the DVM score corresponding to that specific feature set ${{\mathcal {F}}}$. The pseudocode implementing this strategy for DVM-based feature selection is given in Algorithm 2.

### Unsupervised inference

We can extend the definition of DVM for supervised problems to unsupervised clustering models. In the unsupervised problems, we don’t have explicit outcomes to evaluate the model performance.

- **Input:** Input dataset, $\mathbf{X }=\{X_1,...,X_N\}$
  - Labels, $$\mathbf{Y }=\{Y_1,...,Y_N\}$$
  - Desired number of output features, *r*
- $$\mathcal {F}:=\phi $$, $$\mathcal {R}:=\{1,...,r\}$$
- **for** *each* $i\in \mathcal {R}$ **do**
  - $$f \leftarrow _{j\in \mathcal {R}-\mathcal {F}} ( DVM\{\mathcal {F}\} - DVM\{\mathcal {F}\cup X^j\} )$$
  - Add *f* into $$\mathcal {F}$$
- **Output:** $$\mathcal {F}$$

Algorithm 2: DVM-based feature selection.

Intuitively, the definition of *fidelity* for an unsupervised clustering method reflects the stability of the derived clusters, regardless of the clustering labels.

Our strategy for estimating the DVM fidelity for unsupervised clustering methods is based on randomly splitting the dataset $\mathbf{X }$ into three subsets $(\mathbf{X }',\mathbf{X }'',\widetilde{\mathbf{X }})$.

The first two of these sets, $(\mathbf{X }',\mathbf{X }'')$, are used for *cross-validation training*, whereas the remaining one, $\widetilde{\mathbf{X }}$, is used for independent testing and *validation*. By training the classifier on the first subset $(\mathbf{X }')$, we obtain derived computed labels. These predicted labels, ${\widehat{\mathbf{Y }}}$, may be used as baseline for computing the fidelity based on the information bottleneck in equation (12). Let $\widetilde{\mathbf{T }}$ be the representation layer (or predicted indices associated with the test dataset $\widetilde{\mathbf{X }}$). The DVM fidelity term for unsupervised learners may then be defined as follows:

13 $$\begin{aligned} F:= \frac{1}{M}\displaystyle \sum _{i=1}^M \frac{I(\widetilde{\mathbf{T }}_i;\hat{\mathbf{Y }}_i)- \beta I(\widetilde{\mathbf{X }}_i;\widetilde{\mathbf{T }}_i|\hat{\mathbf{Y }}_i)}{I(\mathbf{X };\hat{\mathbf{Y }})}, \end{aligned}$$

where the index *i* in the above definition denote the the variables associated with the *i*th randomized splitting of $\mathbf{X }$. Just as we did for the supervised problems, we can explicate the DVM algorithmic implementation via the *pseudo code* used in the DVM package.

The algorithm below (Algorithm 3) shows the DVM calculation for unsupervised clustering and classification problems. Again, the regularization term is derived using the approximate estimate of the computational complexity associated with the classifier (*R*(*g*)), see Table 1.

- **Input:** Data sets $\mathbf{X }$, $\mathbf{Y }$, model *g*, parameters $\beta$ and $\lambda$
- **for** *a random split* $(\mathbf{X }'_i,\mathbf{X }'', \widetilde{\mathbf{X }}_i)$ *of* $\mathbf{X}$ **do**
  - Apply unsupervised model *g* based on $\widetilde{\mathbf{X }}_i$
  - $$\hat{\mathbf{Y }}_i\leftarrow g(\mathbf{X }'_i)$$
  - $$\widetilde{\mathbf{T }}_i\leftarrow g(\mathbf{X }''_i)$$
  - $$F_i\leftarrow \frac{I(\widetilde{\mathbf{T }}_i;\hat{\mathbf{Y }}_i)- \beta I(\widetilde{\mathbf{X }}_i;\widetilde{\mathbf{T }}_i|\hat{\mathbf{Y }}_i)}{I(\mathbf{X };\hat{\mathbf{Y }})} $$
- $$\widehat{D} \leftarrow \frac{1}{T} \sum _{i=1}^T F_i - \lambda R(g)$$
- **Output**: $$\widehat{D}$$

Algorithm 3: DVM calculation for unsupervised problems.

## Mutual information estimation

In many areas, including data science and machine learning, the density of the data is unknown. In these cases, one needs to estimate the mutual information from the data points. Examples of MI estimation strategies include KSG [40], KDE [41], Parzen window density estimation [42], and adaptive partitioning [43].

The computational complexity and convergence rate are two important performance metrics of various MI estimators. The process of MI estimation is computationally intensive for large data sets, e.g., the computational complexity of the KDE method is $O(n^2)$, while the KSG method takes $O(k\ n \log (n))$ time to compute MI (*k* is a parameter of the KSG estimator). More computationally efficient estimators such as [44] provide improvements with estimated MI estimation time of $O(n \log (n))$.

Thus, estimation of mutual information for large and complex data sets requires some approximation. For instance, we can use one of the standard estimators that exist for the non-parametric distributions. Non-parametric estimators are a family of estimators, for which we consider minimal assumptions on the density functions. There are several previous approaches, e.g., [45–48], that guarantee optimal convergence rates. Among these estimators, the hash-based estimator proposed in [48] has linear computational complexity. As we deal with large and complex data sets, here we employ a hash-based mutual information estimator, called the *ensemble dependency graph estimator (EDGE)* [22]. EDGE has an optimal mean square error (MSE) convergence rate and low computational complexity that make it suitable for our task of detecting the information gain associated with augmenting a data set.

## Results

We conducted a number of experiments to illustrate the use of the proposed DVM on a wide range of real and simulated datasets. Each dataset was labeled as low, medium, or high energy, indicating the strength of the signal information content in the data. The results of different machine learning and statistical modeling methods, their quality, accuracy, and reproducibility heavily depend on the intrinsic signal energy. We contrast the proposed DVM against classifier-accuracy and Bayes optimal classifier accuracy, which is a measure of classification task difficulty. In this paper, we define the Bayes classifier accuracy as the additive complement of the classical Bayes error rate (risk), i.e., *Bayesian Accuracy = 1- Bayesian Error*.

### Datasets

*MNIST Handwritten Digits Data*: The Modified National Institute of Standards and Technology (MNIST) dataset consists of a large number of fixed-size, grayscale images of handwritten digits. It includes a set of 60,000 training images, and a set of 10,000 test images. Each image has a dimension $28\times 28$, and each pixel intensity takes a value between 0 and 255. The training data are also paired with a label (0, 1, 2, ...,9) indicating the correct number represented in the corresponding image [39].

*ALS dataset*: Amyotrophic lateral sclerosis (ALS) is a complex progressive neurodegenerative disorder with an estimated prevalence of about 5 per 100,000 people in the United States. The disease severity is enormous with many the patients surviving only a few years after ALS diagnosis, and few living with ALS for decades [49]. We used the ProACT open-access database [50], which collects and aggregates clinical data of 16 ALS clinical trials and one observational study completed in the recent twenty years [51].

This dataset contains the information of 2,424 patients with 249 clinical features, tracked over 12 months. The ALS disease progression, which is measured by the change of Amyotrophic Lateral Sclerosis Functional Rating Scale (ALSFRS) score over time, is used as the target variable. ALSFSR is a real-valued number in the range [0, 5].

*Simulated dataset*: Synthetic data were generated using *make_blobs* function in scikit-learn (https://scikit-learn.org). There were five centers for the dataset. Each dataset had 2,000 samples and 800 features. The standard deviation for strong signal data was 20, while it was 40 for weak signal data.

The continuous data was generated using the following formula:

14 $$\begin{aligned} Y = X^{\frac{1}{3}} + K\ Noise, \end{aligned}$$

where *X* was generated by sampling 800 random observations from a multivariate Gaussian distribution. The mean vector of this multivariate Gaussian distribution was generated from a Gaussian distribution with mean zero and variance 25. The eigenvalues of the diagonal variance-covariance matrix of the multivariate Gaussian distribution were generated from a *Uniform*(2; 12) distribution. The noise term follows a standard Gaussian distribution and its magnitude term, *K*, was chosen to be 10 for the strong signal or 50 for the weak signal simulated datasets.

### Validation experimental design

**Fig. 2:**
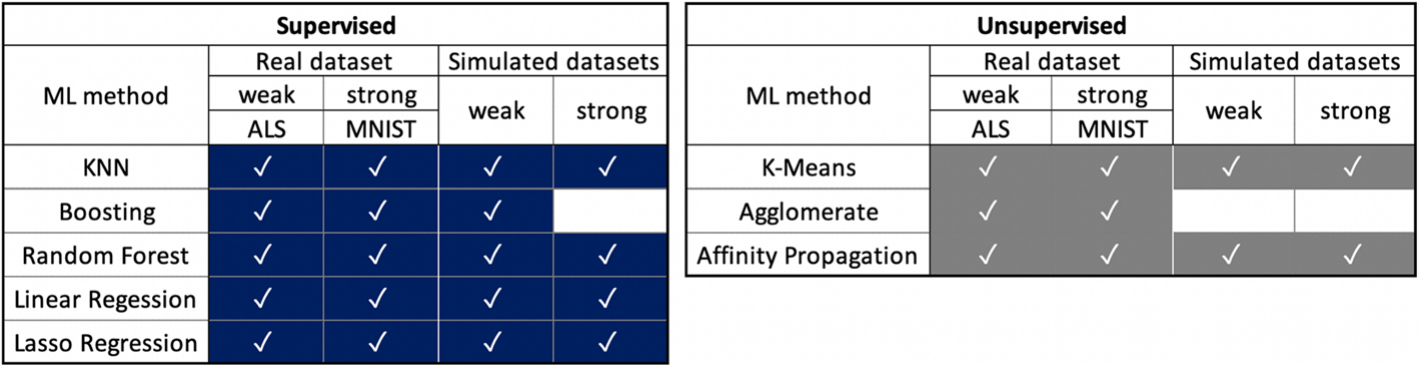
Summary of experimental design

Our experimental design included supervised and unsupervised machine learning methods using real and simulated datasets with different signal profiles – weak and strong signals. Figure 2 shows the specific supervised and unsupervised methods, and the type of data used in the DVM validation protocol. The labels *strong* and *weak* associated with different datasets qualify the relative size of the information content in the data, i.e., the relative signal to noise ratio. For the observed datasets, this information content reflects the power of the covariate features to predict an outcome (for supervised problems) or the consistency of the derived labels (for unsupervised problems). For the simulated data, the information energy is directly related to signal-to-noise ratio ($SNR<0.2$ vs. $SNR>2.0$). For each of the cells in the validation design, we computed the DVM as a parametric surface defined over the 2D grid parameterized over data sample-size and number-of-features. The reported results include 2D plots of cross-sections of the DVM surface for a fixed sample-size or a fixed number-of-features. We also plotted the complete 3D DVM surfaces rendered as triangulated 2-manifolds. These interactive 3D plots are available in supplementary materials and are accessible on our webserver.

**Fig. 3:**
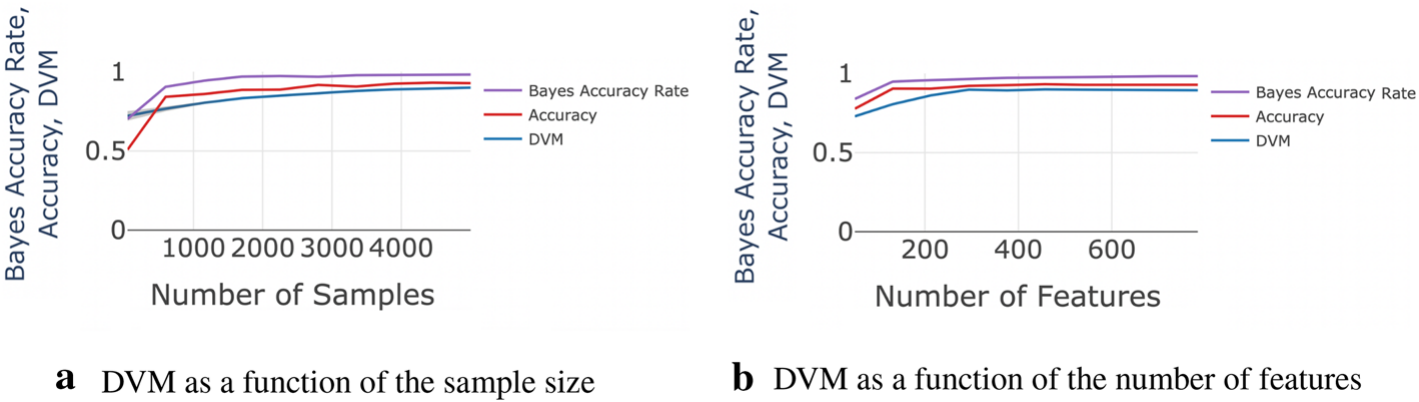
Graph panels (**a, b**) compare the DVM value to the classification accuracy and Bayes accuracy rates for using the Random Forest method on the MNIST dataset, across sample size and the number of features, respectively. As the sample size and the number of features increase, the classification accuracy, Bayes accuracy, and the DVM increase. The shaded area around the DVM curve represents the $95\%$ confidence interval

*Strong signal datasets*: Fig. 3 compares the DVM value to the classification accuracy and Bayes accuracy rates on the MNIST dataset using the Random Forest classifier. As the sample size and the number of features increase, the classification accuracy, Bayes accuracy, and the DVM increase. The $95\%$ confidence interval is represented by the shaded area around the DVM curve.

Using the MNIST data, the results in Fig. 3a imply that both the classification accuracy and DVM drastically increase with increase of the sample size between 500 and 4,500. The accuracy converges to around 0.85 when the sample size approaches 4,500. In the same range, the DVM also converges to around 0.8. Similar results relative to the increase of the number of features are show in Fig. 3b. As the number of features approaches 800, the accuracy converges to around 0.86 and the DVM approaches 0.8.

**Fig. 4:**
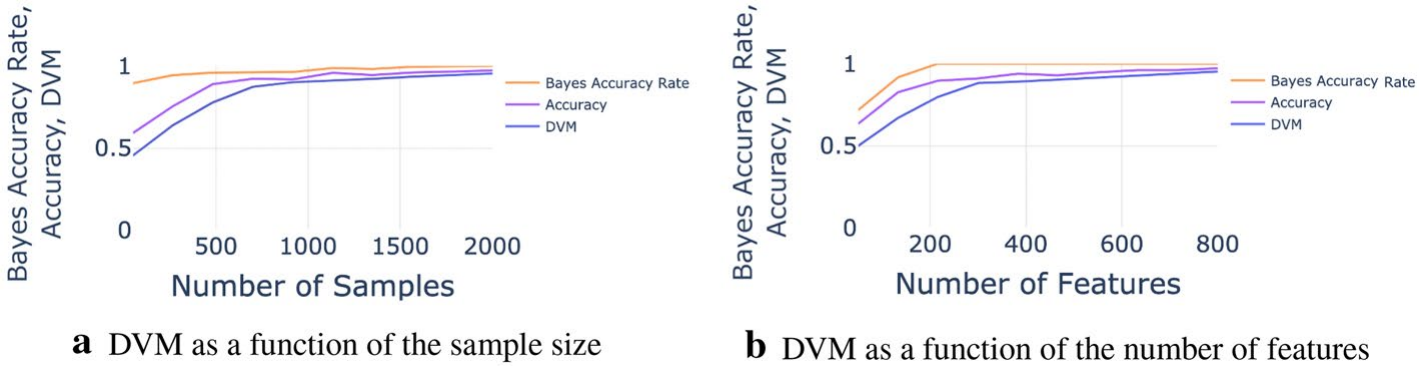
The plots on panels (**a, b**) respectively compare the DVM value to the classification accuracy and Bayes accuracy rates using the Random Forest method on the strong-signal simulated dataset, across sample size and the number of features. As the sample size and the number of features increase, the classification accuracy, Bayes accuracy, and the DVM increase. The shaded area around the DVM curve represents the $95\%$ confidence interval

Using strong-signal simulated data, the results in Fig. 4a show that classification accuracy, Bayes accuracy, and DVM increase as the sample size grows from 200 to 2000. The accuracy converges to around 0.95 and DVM approaches 0.92 for large sample-sizes. The result in Fig. 4b also shows the growth of the classification accuracy and DVM as the number of features increases from 100 to 800, but plateaus around 300 features.

**Fig. 5:**
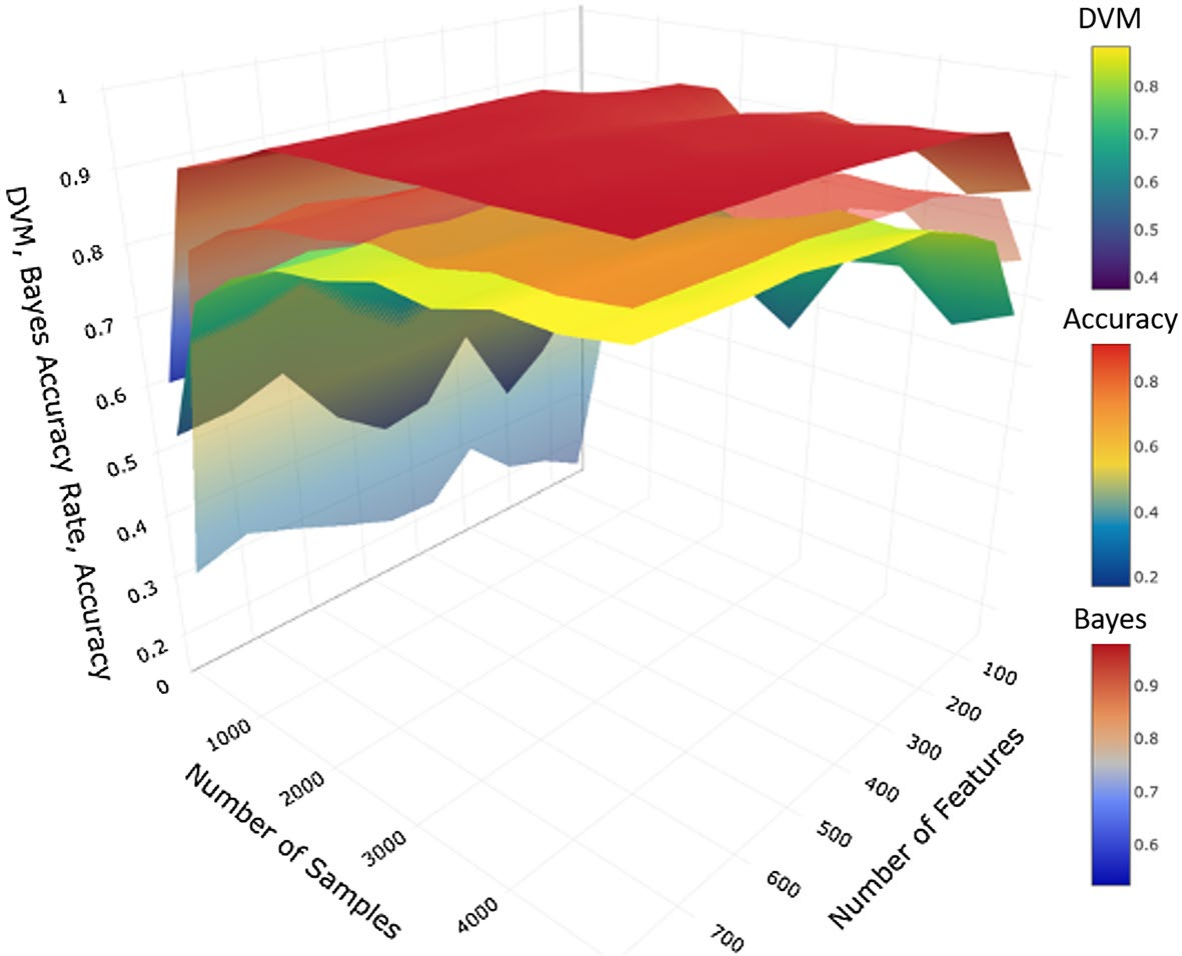
This 3D graph compares the DVM value to the classification accuracy and Bayes accuracy rates for using the K-Nearest Neighbor classifier on the MNIST dataset, in terms of both the number of samples and the number of features

Figure 5 displays a 3D surface plot for the classification accuracy and DVM parameterized by the sample size and the number of features. This graph provides more information compared to the cross-sectional linear plots shown in Figs. 3, 4. Interactive 3D surface plots for all experiments are available online (see Appendix 1, 2).

These results illustrate that for some strong signals, there may be little gain of increasing the sample-size or the number of features.

**Fig. 6:**
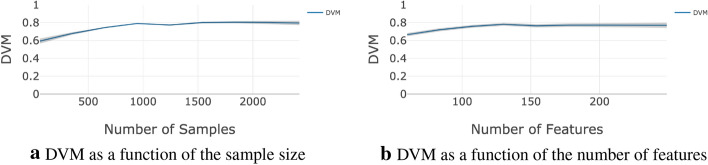
The DVM value in terms of (**a**) sample size (**b**) the number of features, for using the K-Means method on the ALS dataset. The shaded area around the DVM curve represents the $95\%$ confidence interval

*Weak-signal datasets*: Fig. 6 shows the results of the accuracy and DVM for the real (ALS) weak-signal dataset. As expected, the DVM pattern is less stable, but still suggests that adding additional cases or enhancing the features of the data adds little value to improve the unsupervised clustering of the data (K-means clustering).

**Fig. 7:**
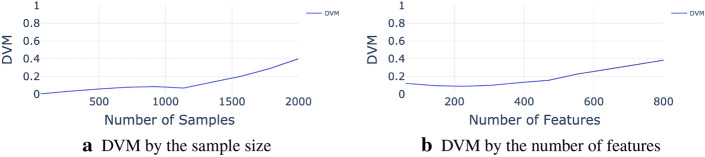
The DVM value in terms of (**a**) sample size (**b**) the number of features, for using the K-Means method on the simulated dataset. The shaded area around the DVM curve represents the 95% confidence interval. The intervals may be too tight and not visible in some plots

Figure 7 depicts the DVM trends using the weak simulated data. Again, the overall low DVM values suggest that increasing the size of augmenting the complexity of weak-signal data may not significantly improve the subsequent unsupervised clustering.

Interactive 2D and 3D DVM surface plots illustrating the results of each experiment are available online at https://socr.umich.edu/docs/uploads/2020/DVM/. These graphs show the behavior of the DVM spanning the domain of possible number of cases and number of features for the real and simulated datasets.

In the appendix, we show examples of cases (pairs of datasets and classifiers) where the DVM may actually decrease with an increase of the number of samples or the number of features.

### Feature selection

**Fig. 8:**
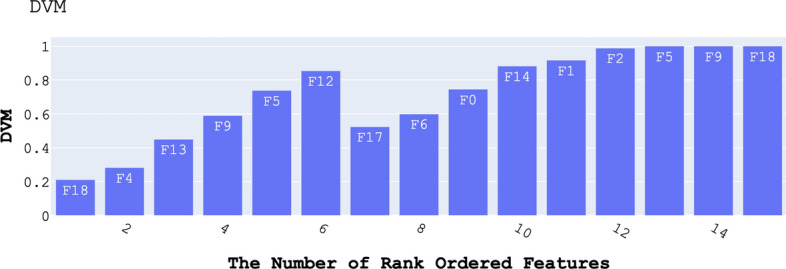
DVM-based feature selection on a simulated dataset. At each step, the best feature that increases the DVM is selected and added to the chosen-features set. Note that due to the dimensionality and runtime complexity terms in the DVM definition, the feature by DVM value graph is not expected to be monotonic. However, the local maxima suggest appropriate stopping criteria for the feature selection algorithm

We demonstrate the feature selection algorithm introduced in algorithm 2 on a simulated dataset. The simulated dataset consists of 1000 samples randomly drawn from a 4-cluster 2D-Gaussian distribution. The clusters are on a square with edge size 1, where the label for each sample determines the distribution cluster. The dimension of the samples is 20 and the problem is to select up to 15 features. Figure 8 represents the steps of the feature selection algorithm. At each step, the best of all features is selected using DVM and added to the chosen-features set. Note that due to the dimensionality and runtime complexity terms in the DVM definition, we do not expect a monotonic graph, however, the local maximums suggest an appropriate stopping criterion for the feature selection process. Figure 8 shows the performance of the DVM-based feature selection yielding a 6-element feature set, $\{F18,F4,F13,F9,F5,F12\}$, corresponding to a high DVM value, $DVM=0.84$.

## Conclusion and discussion

This manuscript presents the mathematical formulation, algorithmic implementation, and computational validation of a data value metric (DVM) for quantifying the analytical-value and information-content (energy) of a dataset. DVM depends on the intended data processing, modeling, forecasting or classification strategies used to interrogate the data. The significance of the work is the introduction of a new measure of intrinsic data value, the DVM, that complements other traditional measures of analytic performance, e.g., accuracy, sensitivity, log-odds ratio, Bayesian risk, positive predictive power, and area under the receiver operating characteristic curve. Through the experiments presented herein, authors discovered that the DVM captures the important trends of traditional measures applied to different types of datasets. The DVM tuning parameter (alpha) provides flexibility for balancing between algorithmic performance and computational complexity, which facilitates a data-specific quantization of the relative information content in a dataset.

As the DVM is applicable for a wide range of datasets and a broad gamut of supervised and unsupervised analytical methods, it can be used as a single unified measure to guide the process of data augmentation, data reduction, and feature selection. It would be interesting to compare the DVM-driven feature selection to other variable selection methods [1], e.g., filtering methods such as information gain and Markov blanket filtering, wrapper techniques such as recursive feature elimination and simulated annealing, and embedded strategies such as random forests and weighted-SVM.

The DVM evaluates the analytical value of a dataset relative to a predefined analytical technique for the data interrogation. The two primary benefits of using an information-theoretic measure, such as the regularized DVM, as a data-utility metric include (1) the estimate of the DVM is easy to compute for each triple of a dataset, analytical strategy, and performance measures, and (2) the DVM magnitude (high or low value) serves as a proxy translating specific data-mining challenges and observable data into a continuous pseudo-distance metric of information-content relative to computational-complexity.

The normalization of the DVM fidelity term ensures that the information-value of the data is standardized in a uniform range, [0,1]. Relative to an a priori analytical strategy, extreme fidelity values close to 0 or 1 correspond respectively to low-quality and high-information-content datasets. The real data and simulation-based results show that there is a connection between *error rate* and the DVM values. However, theoretical bounds on the discrepancy between the prediction error rate and the information-based DVM are not yet understood. Future studies are needed to explore this theoretical relation for various types of analytical methods and data characteristics.

As firm supporters of *open-science*, we have shared all code, data, and results on the DVM GitHub page (https://github.com/SOCR/DVM/) and the SOCR DVM documentation site (https://socr.umich.edu/docs/uploads/2020/DVM/).

## Figures and Tables

**Table 2:**
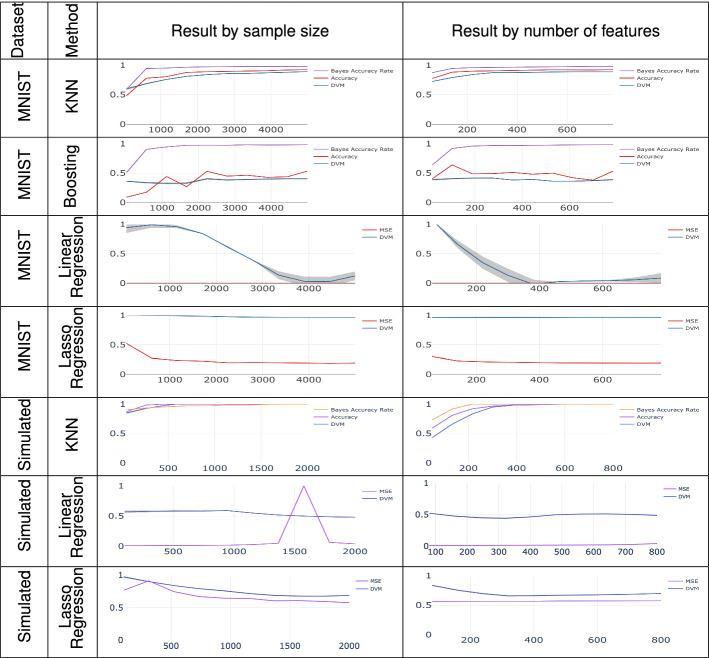
Test results by the signal profile (Supervised - Strong)

**Table 3:**
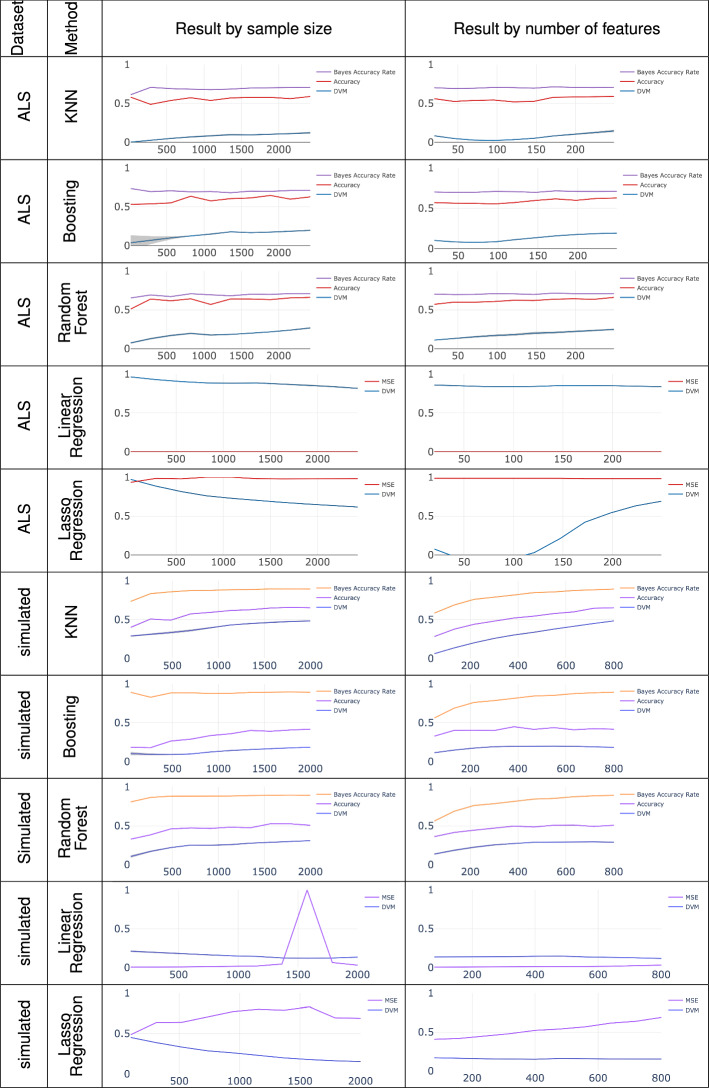
Test results by the signal profile (Supervised - weak)

**Table 4:**
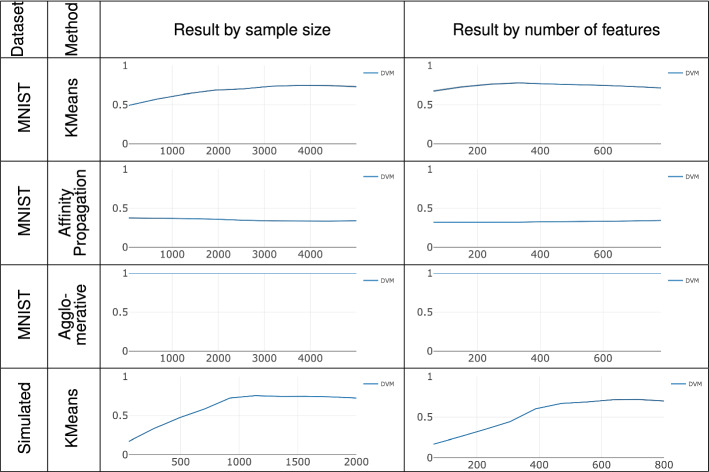
Test results by the signal profile (Unsupervised - Strong)

**Table 5:**
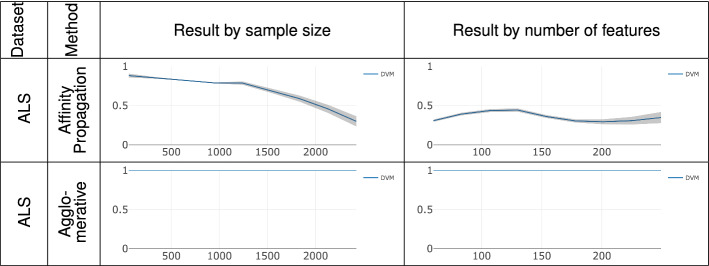
Test results by the signal profile (Unsupervised - Weak)

## Data Availability

All data generated, simulated, or analysed during this study are included in this published article [and its supplementary information files].

## Appendix 1

### Proof of Remark 1

First note that the following equation holds [22]:

15 $$\begin{aligned} I(T;Y|X)=0. \end{aligned}$$

Using the chain rule for the mutual information we have

16 $$\begin{aligned} I(T,X;Y) = I(X;Y)+I(T;Y|X) = I(X;Y). \end{aligned}$$

On the other hand we also have the following equation:

17 $$\begin{aligned} I(T,X;Y) = I(T;Y)+I(X;Y|T). \end{aligned}$$

From (16) and (18) we obtain the following inequality:

18 $$\begin{aligned} I(T,X;Y) = I(X;Y) = I(T;Y)+I(X;Y|T) \ge I(T;Y), \end{aligned}$$

and the equality holds if and only if $I(X;Y|T)=0$. Therefore, $F(1)=1$ if and only if the second term in (6) is equal to zero and we have $I(X;Y|T)=0$. An example of a case with conditions in (9) is when *Y* is an invertible function of *T*. $\square$

## Appendix 2

### Implementation

Below, we briefly describe the DVM Python package organization and invocation. We have implemented a *DVM* python package for our data value metric framework and made it available on GitHub (https://github.com/SOCR/DVM).

The DVM package can be used on any dataset and any user-defined supervised or supervised tasks in order to evaluate the quality of the data. The package consists of three main python files, *DVM.py*, *methods.py*, and *DVM_plot.py*. Please note that *DVM.py* uses the mutual information estimator file, *EDGE.py*, as its dependency.

*DVM.py* gets the input datasets *X* and in the case of a supervised task, a set of corresponding labels denoted by *Y*. Further, the user needs to specify the input parameters, $\beta$, *problem_type* and *method*. $\beta$ is the coefficient of the regularizer term of DVM. *problem_type* specifies whether the task is supervised or unsupervised, and *method* is the learning method that is used by user. For a given method, we can also input the corresponding required arguments. For example, if we are using *KNN_classifier* from *methods.py* as our method, it requires the parameters *n_neighbors* (number of neighbors) and *weights* (type of weights) as input:

$$ \begin{aligned} &{\tt{DVMvalue}}= {\tt{DVM}} ({\tt{X,\ Y,\  problem}}\_{\tt{type}} =\lq{\tt{supervised}}\rq,\\ &\quad {\tt{method}}={\tt{KNN}}\_{\tt{classifier}}, \ {\tt{n}}\_{\tt{neighbors}} = 10, \ {\tt{weights}} = \lq{\tt{uniforms}}\rq)\\ \end{aligned}$$

There are two DVM output values: *DVM_value* and *confidence_band*. *DVM_value* gives the average value computed according to the DVM formula in equation (6) and *confidence_band* gives the $95\%$ confidence limits of the DVM values computed by different subsets in equation (13).

The *methods.py* file consists of various supervised and supervised methods. Each supervised method takes the following arguments: *X_train, Y_train, X_test, **kwargs*. *X_train, Y_train, X_test* respectively are the train data set and labels, and the test data set for which we would like to predict labels. ***kwargs* specifies all of the arguments that the given method requires. An example of the format is as follows:

$$ {\tt{Y}}\_{\tt{predict}}={\tt{KNN}}\_{\tt{classifier}}({\tt X}\_{\tt{train}}, \ {\tt{Y}}\_{\tt{train}}, \ {\tt{X}}\_{\tt{test}}, \ **{\tt{kwargs}})$$

The output of the method is a *numpy* array of predicted labels. Note that in addition to the methods listed in *methods.py* file, any other user defined method that satisfies the above format can be used for DVM.

The *DVM_plot.py* gets the input datasets X, and in the case of a supervised task, a set of corresponding labels denoted by Y. Further the user need to specify the input parameters *continuous*, $\beta$, *problem_type*, *method*, *plot_type*. *Continuous* indicates whether the response variable is continuous variable or discrete variable. *plot_type* specifies the plots that the user wants to generate, where *plot_type* = ’3D’ generates 3D plots of DVM, *plot_type* = ’3D’ generates 2D plots, and *plot_type* = ’Both’ generates both 2D and 3D plots.$\beta$, *problem_type*, *method* have the same meaning as in the *DVM.py*. For a given method, we can also input the corresponding required arguments like *DVM.py*. The same example as *DVM.py* is used here to illustrate the syntax:

In addition to the 2D and 3D DVM plots, *DVM_plot* also outputs a dictionary containing *Accuracy*, *MI*(mutual information), *Complexity*, *DVM*,*Sample Number* (a sequence of different number of samples) and *Feature Number* (a sequence of different of features.)

As calculating the DVM measure actually involves another parameter ($\lambda$) that represents the weight-averaging of the DVM fidelity and regularization terms, the actual DVM manifold is intrinsically a surface embedded in 4D. We have designed a kime-surface visualization that allows us to explore the properties of the DVM manifold by including a $\lambda$ slider that reduces the DVM into an animated 3D space.

### Supplementary experiments

The appendix below includes four additional tables of results that illustrate some of the DVM performance in different situations. Readers are encouraged to view the corresponding DVM interactive 2D plots and 3D surface graphs on the web-site,

https://socr.umich.edu/docs/uploads/2020/DVM/. See Tables 2, 3, 4, 5
